# Elevated triglyceride-to-HDL cholesterol ratio is an indicator for insulin resistance in middle-aged and elderly Taiwanese population: a cross-sectional study

**DOI:** 10.1186/s12944-019-1123-3

**Published:** 2019-10-11

**Authors:** Wei-Chung Yeh, Yu-Chung Tsao, Wen-Cheng Li, I-Shiang Tzeng, Liang-Sien Chen, Jau-Yuan Chen

**Affiliations:** 1 Department of Family Medicine, Chang-Gung Memorial Hospital, Linkou Branch, Postal address: No. 5, Fuxing St., Guishan Dist, Taoyuan City, 33305 Taiwan, Republic of China; 2 Chang Gung University College of Medicine, No.259, Wenhua 1st Rd., Guishan Dist., Taoyuan City, 333 Taiwan, Republic of China; 3 Department of Occupational Medicine, Chang Gung Memorial Hospital, Linkou Branch, No.5, Fuxing St., Guishan Dist., Taoyuan City, 33305 Taiwan, Republic of China; 4 Department of Health Management, Xiamen Chang-Gung Hospital, Xiamen, No.123, Xiafei Road, Haicang District, Xiamen, China; 5 Department of Research, Taipei Tzu Chi General Hospital, Buddhist Tzu Chi Medical Foundation, No.289, Jianguo Rd., Xindian Dist, New Taipei City, 23142 Taiwan, Republic of China

**Keywords:** Anthropometry, Body fat; insulin resistance, Prediction, Triglyceride to high-density lipoprotein cholesterol ratio

## Abstract

**Background:**

Previous studies have reported that the triglyceride to high-density lipoprotein cholesterol (TG/HDL-C) ratio could be a simple clinical indicator of insulin resistance (IR), but the results indicated that there were heterogeneities between different ethnicities. We aimed to investigate the association between TG/HDL-C and IR (as measured by homeostasis model assessment of IR [HOMA-IR]), and establish a clinical prediction rule for IR in middle-aged and elderly Taiwanese.

**Methods:**

A total of 398 subjects were recruited, and each subject completed a questionnaire that included personal and medical history data, and underwent anthropometric measurement and blood sampling. IR was defined as HOMA-IR index value ≥2.0. Chi-squared test, independent two-sample t-test, Pearson’s correlation coefficient, and multiple logistic regression were used to evaluate the association between IR and TG/HDL-C ratio. A receiver operating characteristic (ROC) analysis was conducted to evaluate the ability of the developed clinical prediction rule to correctly discriminate between subjects of IR positive and IR negative groups.

**Results:**

A significant association between IR and TG/HDL-C ratio was identified with a Pearson’s correlation coefficient of 0.35 (*p*-value< 0.001). In multiple logistic regression, high BMI (OR = 1.23; 95% C.I. = 1.13–1.33), hypertension (OR = 1.90; 95% C.I. = 1.12–3.21), diabetes mellitus (OR = 5.44; 95% C.I. = 2.93–10.08) and high TG/HDL ratio (OR = 1.45; 95% C.I. = 1.23–1.72) were significantly associated with the risk of elevated HOMA-IR.

The area under ROC curves for TG/HDL-C ratio was 0.729 and the optimal threshold value was 2.197 where the corresponding of sensitivity and specificity were 72.4 and 65.1%.

**Conclusions:**

Our findings showed that the elevated TG/HDL-C ratio was significantly associated with IR and could be used as an indicator of IR among the middle-aged and elderly population in Taiwan. It is clinically available, thus eliminating any additional costs. Future research is warranted to investigate the use of TG/HDL-C ratio combined with other risk factors for predicting IR under diverse ethnic backgrounds.

## Background

Insulin resistance (IR), the most common feature of obesity, is a major pathophysiological factor in the development and progression of diabetes mellitus (DM) and cardiovascular disease [1–4]. Currently, several directly and indirectly methods for assessing IR are available [5]. Among them, the gold standard method is the hyperinsulinemic euglycemic clamp test (HEC test), originally developed by DeFronzo [6]. However, the method is expensive, invasive, and time-consuming, making it not feasible for either epidemiological investigations or routine clinical applications [7]. Therefore, the homeostasis model assessment of IR (HOMA-IR) has emerged to evaluate IR [6, 8].

The lack of standardized insulin assay has limited the clinical utility of HOMA-IR, although it has been widely used in the study of metabolic syndrome [9]. Additionally, the HOMA-IR is not routinely measured in clinical practice when used for quantifying IR. In a clinical perspective, a simple and more accessible marker for predicting IR can be useful for early identification of subjects with IR for clinicians and eliminate any additional costs.

Some evidences have indicated that hypertriglyceridemia and low high-density lipoprotein cholesterol are two key metabolic abnormalities associated with IR states [10, 11]. Previous studies have shown that the triglyceride to high-density lipoprotein cholesterol (TG/HDL-C) concentration ratio is closely related to IR [12, 13]. In these studies, TG/HDL-C ratio has been identified as a simple clinical indicator of IR and a predictor of diabetes [14] and coronary heart disease [15].

Recently, the TG/HDL-C ratio has been investigated for various potential clinical uses in adult and pediatric populations [13, 16–26]. Thus, it seems to help clinicians identify IR persons by using the TG/HDL-C ratio, based on commonly available and standardized measurements. While using the TG/HDL-C ratio to identify IR, it should be noted that the relationship between TG/HDL-C and insulin may differ by ethnicity. It means that the results may be heterogeneity between different ethnicities. Hence, using TG/HDL-C to predict IR would not be appropriate in certain populations [27, 28]. Moreover, few studies have predicted IR by using TG/HDL-C among a middle-aged and elderly population in Taiwan**.**

This study was conducted to investigate the association between TG/HDL-C and IR (as measured by HOMA-IR) and to establish a clinical prediction rule for IR through the marker, TG/HDL-C ratio, among a middle-aged and elderly population in Taiwan.

## Methods

### Study design and study population

Participants in this study were recruited from a community health promotion project of Linkou Chang Gung Memorial Hospital between February 2014 and August 2014. In total, 400 participants aged 50 years or over were recruited through posters and advertisements from the community office.

This study was approved by the Chang Gung Medical Foundation Institutional Review Board and written informed consent form was given by all participants before enrollment. A questionnaire that included personal information and medical history was completed through a face-to-face interview for each participant. Participants with incomplete or missing data were excluded. The final number of participants was 398.

### Anthropometric and laboratory examinations

Information on demographic including age, sex was collected after obtaining informed consent. WC was measured at the level midway between the iliac crest and the lower border of the 12th rib. Body mass index (BMI) was calculated as weight divided by the square of the height (kg/m^2^).

Blood sampling were conducted by trained nurses. Participants were requested to fast for a minimum of 12 h and to avoid a high-fat diet or alcohol consumption before blood sampling. Venous blood samples were stored in a refrigerator at 4 °C prior to analysis in the hospital laboratory. TG, total cholesterol, HDL-C, low-density lipoprotein cholesterol (LDL-C), glucose, insulin, fasting plasma glucose (FPG), and alanine aminotransferase (ALT) were measured using blood sample collected from each participant in a hospital laboratory accredited by the College of American Pathologists.

### Diagnosis of insulin resistance

HOMA-IR was calculated by fasting glucose (in mmol/L) × fasting insulin (in mU/ml)/22.5 [29]. In a study of 2649 Chinese subjects, the HOMA-IR cut-off value to identify diabetes was 1.97 as determined by the receiver-operating characteristic (ROC) curve, and 2.03 in the 90th percentile of subjects with normal glucose tolerance [30].

Because most Taiwanese ancestors emigrated from China hundred years ago, we defined IR as ≥2 in our study.

### Statistical analysis

Statistical analyses were performed using SPSS version 22 software (IBM, SPSS Armonk, NY, IMM Corp). A two-sided *p*-value < 0.05 was considered as statistical significance. Participants were divided into IR positive and IR negative groups using a cut-off value of 2. Continuous variables were expressed as mean and standard deviation, and categorical variables were represented by counts and percentages. Differences in demographic and clinical characteristics of participants in each group were compared using independent two-sample t-test for continuous data and chi-square test for categorical data. Pearson’s correlation coefficient was calculated for the correlations between cardiovascular disease risk factors and IR.

Multiple logistic regression analysis was conducted to evaluate other covariates that were significantly associated with IR in addition to TG/HDL-C. A ROC analysis was conducted to evaluate the ability of the established model for correctly discriminating the participants of IR positive and IR negative. The ROC curves for the established model was made and the overall diagnostic accuracy was quantified using the area under the ROC curve (AUC). The optimal cut-off points were determined by the Youden’s index, and the corresponding sensitivity and specificity were calculated.

## Results

### Descriptive statistics

A total of 400 participants were recruited through posters and advertisements from the community office. Two people were excluded for extreme values, incomplete or missing data. Finally, 398 participants were enrolled in this study.

Table 1 shows the general demographic and characteristics of participants according to IR positive and IR negative that categorized by HOMA-IR using a cut-off value of 2. The subjects comprised of 139 males (35%) and 259 females (65%), with an overall mean age of 64.43 ± 8.45 years. The average BMI and WC were 24.54 ± 3.57(kg/m^2^) and 84.99 ± 9.62 cm, respectively. The mean HOMA-IR index was 1.86 ± 1.39 and the mean TG/HDL-C ratio was 2.52 ± 1.83. There were no significantly differences in age, creatinine, percentage of current smoking, alcohol drinking, and vegetarian between IR positive and IR negative groups. The IR positive group had higher blood pressure, BMI, waist circumference, fasting glucose and TG/HDL-C ratio than the IR negative group.

**Table 1:** General characteristics of the study population according to IR positive and IR negative

Variables	Insulin Resistance						
	Total		IR positive		IR negative		
	(*n* = 398)		(*n* = 123)	(≧2)	(*n* = 275)	(< 2)	*p*-value
Age (year)	64.43	±8.45	64.59	±7.86	64.36	±8.71	0.80
SBP (mmHg)	129.57	±16.72	133.97	±16.49	127.60	±16.47	< 0.001
DBP (mmHg)	76.94	±11.36	78.91	±10.66	76.06	±11.57	0.02
BMI (kg/m^2^)	24.54	±3.57	26.43	±3.69	23.70	±3.18	< 0.001
Waist circumference (cm)	84.99	±9.62	90.51	±9.86	82.53	±8.43	< 0.001
ALT (U/L)	22.66	±12.97	27.28	±17.01	20.60	±10.06	< 0.001
Creatinine (mg/dL)	0.77	±0.42	0.78	±0.33	0.77	±0.45	0.88
eGFR (ml/min/1.73m^2^)	113.25	±33.21	109.95	±31.90	114.73	±33.73	0.19
FPG (mg/dL)	95.54	±22.32	110.06	±31.81	89.05	±11.59	< 0.001
HDL-C (mg/dL)	54.51	±13.91	48.68	±11.37	57.12	±14.17	< 0.001
HOMA-IR index	1.86	±1.39	3.39	±1.58	1.18	±0.44	< 0.001
LDL-C (mg/dL)	118.47	±32.16	112.11	±29.83	121.31	±32.80	0.01
Total Cholesterol (mg/dL)	197.19	±35.69	191.46	±34.08	119.75	±36.16	0.03
TG/HDL	2.52	±1.83	3.48	±2.32	2.09	±1.37	< 0.001
Triglyceride (mg/dL)	121.20	±62.93	153.46	±76.04	106.77	±49.88	< 0.001
Uric Acid (mg/dL)	5.74	±1.41	6.10	±1.36	5.58	±1.41	0.001
WBC (1000/uL)	6.04	±1.59	6.71	±1.79	5.74	±1.39	< 0.001
Men, n(%)	139	(34.9)	41	(33.3)	98	(35.6)	0.66
Current smoking, n(%)	42	(10.6)	12	(9.8)	30	(10.9)	0.73
Alcohol drinking ≧2 times/week, n(%)	75	(18.8)	17	(13.8)	58	(21.1)	0.09
Regular exercise, n(%)	326	(81.9)	92	(74.8)	234	(85.1)	0.01
Vegetarian, n(%)	22	(5.5)	5	(4.1)	17	(6.2)	0.39
ACR ≧30 mg/g, n(%)	74	(18.6)	31	(25.2)	43	(15.6)	0.02
HTN, n(%)	199	(50.0)	84	(68.3)	115	(41.8)	< 0.001
DM, n(%)	77	(19.3)	48	(39.0)	29	(10.5)	< 0.001
Hyperlipidemia, n(%)	259	(65.1)	93	(75.6)	166	(60.4)	0.003
Metabolic syndrome	141	(35.4)	89	(72.4)	52	(18.9)	< 0.001

Notes: Clinical characteristics are expressed as mean ± SD for continuous variables and n (%) for categorical variables. P-value were derived from independent two-sample t-test for continuous variables and chi-square test for categorical variables

Abbreviations: *SBP* systolic blood pressure; *DBP* diastolic blood pressure; *BMI* body mass index; *eGFR* estimated glomerular filtration rate; *FPG* fasting plasma glucose; *HDL-C* high-density lipoprotein cholesterol; *LDL-C* low-density lipoprotein cholesterol; *WBC* white blood cell count; *ACR* albumin to creatinine ratio; *HTN* hypertension; *DM* diabetes mellitus

### Correlation analysis

**Fig. 1:**
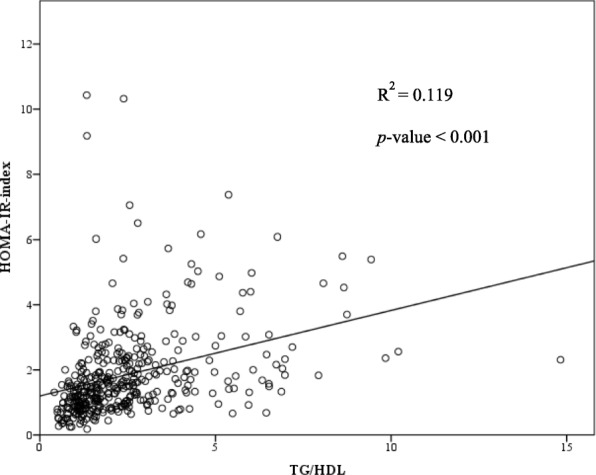
Representation of the correlation analysis: there was a trend toward a positive correlation between HOMA-IR-index and TG/HDL

Table 2 shows the correlations between different anthropometric indices and HOMA-IR with and without adjusting for age. The variables including BMI, waist circumference, fasting glucose, TG, and TG/HDL-C were statistically significantly positively associated with HOMA-IR before and after adjusting for age. The variable, HDL-C, was statistically significantly negatively associated with HOMA-IR. Figure 1 demonstrates the positive correlation between TG/HDL-C and HOMA-IR index with a Pearson’s correlation coefficient of 0.35.

**Table 2:** The correlation between HOMA-IR index and cardiovascular disease risk factors

Variables	HOMA-IR index (*n* = 398)			
	Unadjusted		Adjusted for age	
	Pearson’s coefficient	*p*-value	Pearson’s coefficient	*p*-value
Age (year)	0.02	0.70	NA	NA
SBP (mmHg)	0.17	0.001	0.17	0.001
DBP (mmHg)	0.09	0.079	0.10	0.06
BMI (kg/m^2^)	0.43	< 0.001	0.433	< 0.001
Waist circumference (cm)	0.41	< 0.001	0.41	< 0.001
FPG (mg/dL)	0.50	< 0.001	0.50	< 0.001
HDL-C (mg/dL)	−0.31	< 0.001	−0.31	< 0.001
LDL-C (mg/dL)	−0.10	0.06	−0.09	0.06
TG (mg/dL)	0.33	< 0.001	0.33	< 0.001
TG/HDL	0.35	< 0.001	0.35	< 0.001

Abbreviations: *SBP* systolic blood pressure; *DBP* diastolic blood pressure; *BMI* body mass index; *FPG* fasting plasma glucose; *HDL-C* high-density lipoprotein cholesterol; *LDL-C* low-density lipoprotein cholesterol; *TG* triglyceride

### Multivariable analysis

Table 3 shows the four models of the association between TG/HDL-C and HOMA-IR index adjusting for related covariates through multiple logistic regression analyses. In Model 1, the odds ratios (OR) were calculated after adjusting for sex. In Model 2, the OR were calculated after adjusting for sex, age and BMI. In Model 3, we examined the relationship between TG/HDL and IR after adjusting for additional confounding variables such as hypertension (HTN), DM and hyperlipidemia. In Model 4, we adjusted for sex, age, BMI, current smoking status, HTN, DM and hyperlipidemia. In Model 1, an increase of 1 unit in the TG/HDL-C ratio was associated with an elevation in the IR by 59%, while in Model 2–4, the corresponding in the TG/HDL-C ratio was associated with 48, 45, and 45% elevation in the odds, respectively. In Model 4 (the final model), BMI (adjusted OR [aOR]: 1.23; 95% confidence interval [C.I.]: 1.13–1.33; *p*-value< 0.001), HTN (aOR: 1.90, 95% C.I.: 1.12–3.21; *p*-value < 0.05), DM (aOR: 5.44, 95% C.I.: 2.93–10.08; *p*-value < 0.001), and TG/HDL-C (aOR: 1.45, 95% C.I.: 1.23–1.72; *p*-value < 0.001) were all significantly associated with HOMA-IR index.

**Table 3:** Association between TG/HDL levels and HOMA-IR index

Variables	Odds ratio	95% C.I.	*p*-value
Model 1			
Sex (men versus women)	0.67	0.41–1.10	0.11
TG/HDL	1.59	1.38–1.83	< 0.001
Model 2			
Sex (men versus women)	0.59	0.34–1.00	0.05
Age (year)	1.01	0.98–1.04	0.52
BMI (kg/m^2^)	1.24	1.15–1.34	< 0.001
TG/HDL	1.48	1.28–1.72	< 0.001
Model 3			
Sex (men versus women)	0.59	0.34–1.04	0.07
Age (year)	0.99	0.96–1.02	0.58
BMI (kg/m^2^)	1.23	1.13–1.33	< 0.001
HTN (yes versus no)	1.88	1.11–3.18	0.02
DM (yes versus no)	5.41	2.93–10.01	< 0.001
Hyperlipidemia (yes versus no)	0.95	0.53–1.70	0.85
TG/HDL	1.45	1.23–1.72	< 0.001
Model 4			
Sex (men versus women)	0.64	0.36–1.15	0.14
Age (year)	0.99	0.96–1.02	0.51
BMI (kg/m^2^)	1.23	1.13–1.33	< 0.001
Smoking (yes versus no)	0.68	0.26–1.77	0.42
HTN (yes versus no)	1.90	1.12–3.21	0.02
DM (yes versus no)	5.44	2.93–10.08	< 0.001
Hyperlipidemia (yes versus no)	0.97	0.54–1.74	0.91
TG/HDL	1.45	1.23–1.72	< 0.001

Abbreviations: *BMI* body mass index; *HDL-C* high-density lipoprotein cholesterol; *TG* triglyceride; *HTN* hypertension; *DM* diabetes mellitus; *CI* confidence interval

### ROC analysis

**Fig. 2:**
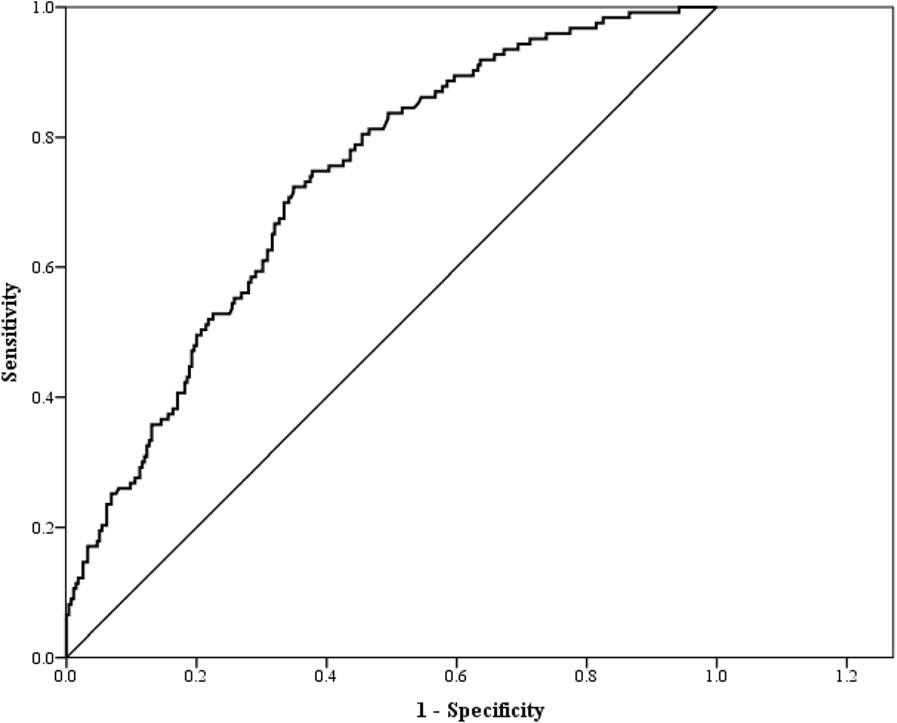
ROC curve for TG/HDL as a predictor of HOMA-IR index

Figure 2 shows the ROC curve for TG/HDL-C as a predictor for HOMA-IR index. The AUC of TG/HDL-C for predicting HOMA-IR index was 0.729, which was significant, with a *p*-value lower than 0.001, as shown in Table 4. The optimal cut-off point calculated by the Youden’s index for using the TG/HDL-C ratio to predict HOMA-IR index was 2.197 where the corresponding sensitivity and specificity were 72.4 and 65.1%, respectively.

**Table 4:** The areas under ROC curve (AUC), sensitivity, specificity by the optimized cut-off points for TG/HDL in predicting HOMA-IR index

Variables	AUC	*p*-value	Cut-off	Sensitivity	Specificity
TG/HDL	0.729	< 0.001	2.197	0.724	0.651

Abbreviations: *HDL-C* high-density lipoprotein cholesterol; *TG* triglyceride; *ROC curve* receiver operating characteristic curve; *CI* confidence interval

## Discussion

The current findings of the study indicate that the TG/HDL-C ratio is strongly associated with IR (as measured by HOMA-IR) in middle-aged and elderly Taiwanese. A high value of TG/HDL-C ratio was positively related to HOMA-IR after adjusting for potential confounding variables. TG/HDL-C ratio was significantly higher in subjects with HOMA-IR index ≥2 (IR positive) as compared to those with HOMA-IR index< 2 (IR negative). The results showed that using the TG/HDL-C ratio to detect IR was valid.

The mechanism of high TG and low HDL is adipose tissue trap and retains less fatty acid in IR status. Thus, increased free fatty acid was transported to liver to synthesize more TG and TG containing very low-density lipoproteins (VLDL). Besides, the TG of TG containing VLDL and the cholesteryl ester of HDL exchange as the plasma TG concentration increases. This action forms the TG-rich HDL that is easily catabolized. Therefore, high TG, high TG/HDL, and low HDL were observed in IR patients [31].

Additionally, the AUC value of the TG/HDL-C ratio to predict IR (as measured by HOMA-IR) was 0.729, where AUC ≥ 0.7 is generally considered acceptable test performance. The optimal threshold was 2.197 with a corresponding sensitivity and specificity of 72.4 and 65.1%. We considered that TG/HDL-C is an acceptable marker that can be used alone for IR assessment in middle-aged and elderly Taiwanese. A study of 812 Taiwanese adults [32] reported that the AUC of the models including TG/HDL-C ratio, sex, greater waist circumferences, and higher ALT levels was 0.71 without cut-off set. The performance of their models was similar to ours, and they were all conducted in Taiwanese adults.

Previous studies had reported different cut-off values of TG/HDL-C ratio to detect the presence of IR. The results of the cut-off values were inconsistent among different ethnic groups. A cross-sectional study of 258 subjects was the first to suggest using the TG/HDL-C ratio as a predictor of IR. In that study, it reported that a cut-off value of TG/HDL-C ratio of 3.0 could reliably predict IR in overweight people [13]. Subsequently, in another cross-sectional cohort of 90 overweight African Americans [33], the TG/HDL-C ratio was tested and the AUC value of the TG/HDL-C ratio to predict IR was 0.56. Consequently, that study did not support using the TG/HDL-C ratio as a predictor of IR in African Americans, because the relationship between TG/HDL-C and insulin may differ by ethnicities. This result reinforces the motivation of this study.

The Pearson’s correlation coefficient showed that the covariates (BMI, WC, FPG, HDL-C, TG and TG/HDL-C) are all significantly associated with IR (as measured by HOMA-IR) regardless of age. After adjusting for covariates such as age, sex, BMI, current smoking status, HTN, DM, and hyperlipidemia, TG/HDL-C remained significantly associated with IR (as measured by HOMA-IR) (Table 3). This result reinforces the relationship between IR and TG/HDL-C ratio. Therefore, we suggested that using TG/HDL-C ratio as an index for early identification of IR in middle-aged and elderly Taiwanese.

Several limitations were associated with our study. First, some information that related to IR were not recorded, such as the detail of regular physical exercise and family history of diabetes in the study population. Second, this study was a cross-sectional design that made it difficult for us to explore the causal relationship between TG/HDL-C and IR. Third, the accuracy of the models need additional evaluation when applied to other ethnic groups because ethnicity affects the associations between TG/HDL-C and IR.

## Conclusions

In conclusion, our findings showed that the elevated TG/HDL-C ratio was significantly associated with IR and could be used as an indicator of IR among the middle-aged and elderly population in Taiwan. It is clinically available, thus eliminating any additional costs. Future research is warranted to investigate the use of TG/HDL-C ratio combined with other risk factors related to IR for predicting IR under diverse ethnic backgrounds.

## Data Availability

The datasets used and/or analyzed during the current study are available from the corresponding authors upon reasonable request.

## Abbreviations

ALT: Alanine aminotransferase
BMI: Body Mass Index
BP: Blood pressures
CRP: C-reactive protein
DM: Diabetes mellitus
FPG: Fasting plasma glucose
HDL-C: High-density lipoprotein cholesterol
HOMA-IR: Homeostasis model assessment of insulin resistance
IR: Insulin resistance
LDL-C: Low-density lipoprotein cholesterol
TG: Triglyceride
TG/HDL-C: Triglyceride to high-density lipoprotein cholesterol
ROC: Receiver operating characteristic
AUC: Area under the ROC curve
HTN: Hypertension
aOR: Adjusted odds ratio
IDF: International Diabetes Federation.
WBC: White blood cell count
WC: Waist Circumference
